# Supplementary material for: Germline variation in ADAMTSL1 is associated with prognosis following breast cancer treatment in young women
Source: Nat Commun. 2017 Nov 21;8:1632. doi: 10.1038/s41467-017-01775-y (PMC5696339; doi:10.1038/s41467-017-01775-y)
Supplement: Supplementary file 1 — Supplementary Information [file 41467_2017_1775_MOESM1_ESM.pdf]

Supplementary Table 1. Quality control of SNPs and patients

Quality control measure	Stage-1				Stage-2
	**ABCFS**	**HEBCS**	**POSH**	**SUCCESS-A**	**POSH**
Observed SNPs pre QC	536,545	561,466	559,348	693,543	95
Failed genotyping	0	0	0	0	12
Observed SNPs with MAF ≤5%	28,022	58,843	54,872	113,088	5
Observed SNPs with ≥10% missing genotypes	0	387	739	12,225	0
Observed SNPs with HWE *P*-value ≤ $1x10^{-10}$	18	354	169	1,585	2
Observed SNPs passing QC	508,505	501,822	503,568	566,645	76
Imputed SNPs passing QC	5,150,529	5,395,529	5,196,034	5,006,474	NA
Total patients pre QC	214	832	574	3,277	1303
Patients with ≥10% missing genotypes	0	6	2	0	0
Patients with gender mismatch	0	0	0	0	0
Patients with relatedness	0	0	0	1	0
Patients with non-European ethnicity	12	2	16	93	0
Patients with incomplete phenotyping	0	26	0	0	0
Patients remaining	202	798	556	3,183	1303

QC: quality control, MAF: minor allele frequency, HWE: Hardy-Weinberg equilibrium

Supplementary Table 2. The relationship between age of onset and prognosis

SNP		All cohorts (n=6020) Event status		All cohorts, onset age ≤40 (n=2293) Event status		All cohorts, onset age >40 (n=3514) Event status		HEBCS and SUCCESS-A onset age ≤40 (n=456) Event status	
		**0**	**1**	**0**	**1**	**0**	**1**	**0**	**1**
	**AA**	2400	644	832	303	1494	305	199	46
	**CA**	1891	595	653	318	1178	249	146	31
**rs715212**	**CC**	353	137	110	77	233	55	19	15
**DFS**	**MAF**	0.280	0.316	0.274	0.338	0.283	0.295	0.253	0.332
	**Allelic Chi-square *P*-value (OR)**	0.0002 (1.19)		1.03×10^{-5} (1.36)		0.4074 (1.06)		0.0313 (1.47)	
	**Survival meta-analysis *P*-value (OR)**	0.0041 (1.13)		3.54×10^{-5} (1.27)		0.7757 (0.98)		0.0418 (1.37)	
	**GG**	2445	657	839	300	1527	322	195	39
	**CG**	1815	585	629	320	1136	235	143	45
**rs10963755**	**CC**	343	119	129	69	200	47	22	8
**DFS**	**MAF**	0.272	0.302	0.278	0.332	0.268	0.272	0.260	0.332
	**Allelic Chi-square *P*-value (OR)**	0.0017 (1.16)		1.98×10^{-4} (1.29)		0.0853 (1.02)		0.0514 (1.41)	
	**Survival meta-analysis *P*-value (OR)**	0.0147 (1.11)		3.91×10^{-4} (1.22)		0.6834 (0.97)		0.0898 (1.32)	
	**AA**	3975	1122	1380	575	2461	490	300	70
	**AG**	650	245	212	119	428	114	59	20
**rs12302097**	**GG**	12	23	7	7	16	5	5	2
**DFS**	**MAF**	0.073	0.105	0.071	0.095	0.079	0.102	0.095	0.130
	**Allelic Chi-square *P*-value (OR)**	4.94×10^{-8} (1.49)		0.00486 (1.38)		0.0093 (1.32)		0.1533 (1.43)	
	**Survival meta-analysis *P*-value (OR)**	7.54×10^{-5} (1.30)		6.77×10^{-5} (1.45)		0.0742 (1.19)		0.1057 (1.41)	
	**TT**	4152	882	1436	475	2597	352	332	45
	**CT**	698	173	238	100	445	65	63	12
**rs410155**	**CC**	35	8	12	6	23	2	2	0
**OS**	**MAF**	0.079	0.089	0.078	0.096	0.080	0.082	0.084	0.105
	**Allelic Chi-square *P*-value (OR)**	0.1139 (1.14)		0.0458 (1.27)		0.8229 (1.03)		0.4595 (1.28)	
	**Survival meta-analysis *P*-value (OR)**	1.28×10^{-4} (1.34)		0.0049 (1.32)		0.0066 (1.32)		0.5613 (1.20)	

Genotypes for each of the SNPs are presented in the order common homozygous, heterozygous and rare homozygous. Event status is coded 0 for no event and 1 for event present. DFS: disease-free survival, OS: overall survival. Allelic chi-square *P*-value is for the association between the risk allele frequency and event status.

Supplementary Table 3. Association with triple negative breast cancer

Analysis	SNP	All patients			Patients with TNBC		
		Events/N	$P_{univariate}$	HR (CI)	Events/N	$P_{univariate}$	HR (CI)
Early onset DFS	rs715212	698/2293	1.94×10^{-5}	1.28 (1.14-1.43)	152/464	0.004	1.43 (1.12-1.82)
Early onset DFS	rs10963755	689/2286	6.50×10^{-4}	1.21 (1.09-1.36)	148/462	0.141	1.20 (0.94-1.52)
All patients DFS	rs12302097	1312/5825	1.70×10^{-4}	1.28 (1.13 – 1.46)	226/903	0.032	1.45 (1.03-2.05)
All patients OS	rs410155	1063/5948	0.014	1.22 (1.04 – 1.44)	183/884	0.004	1.60 (1.16-2.21)

Univariate Cox regression analyses of triple negative breast cancer patients (TNBC) of the pooled data from HEBCS, POSH (stages 1 and 2) and SUCCESS-A for DFS (disease-free survival: local recurrence, distant metastasis or death from any cause) or OS (death from any cause). The ABCFS cohort was excluded from the analysis due to unavailability of hormone data. The hazard ratios (HRs) and 95% confidence intervals (CI) are presented for the four most significant SNPs.

Supplementary Figure 1. Multi-dimensional scaling (MDS) plot for identifying ethnic outliers

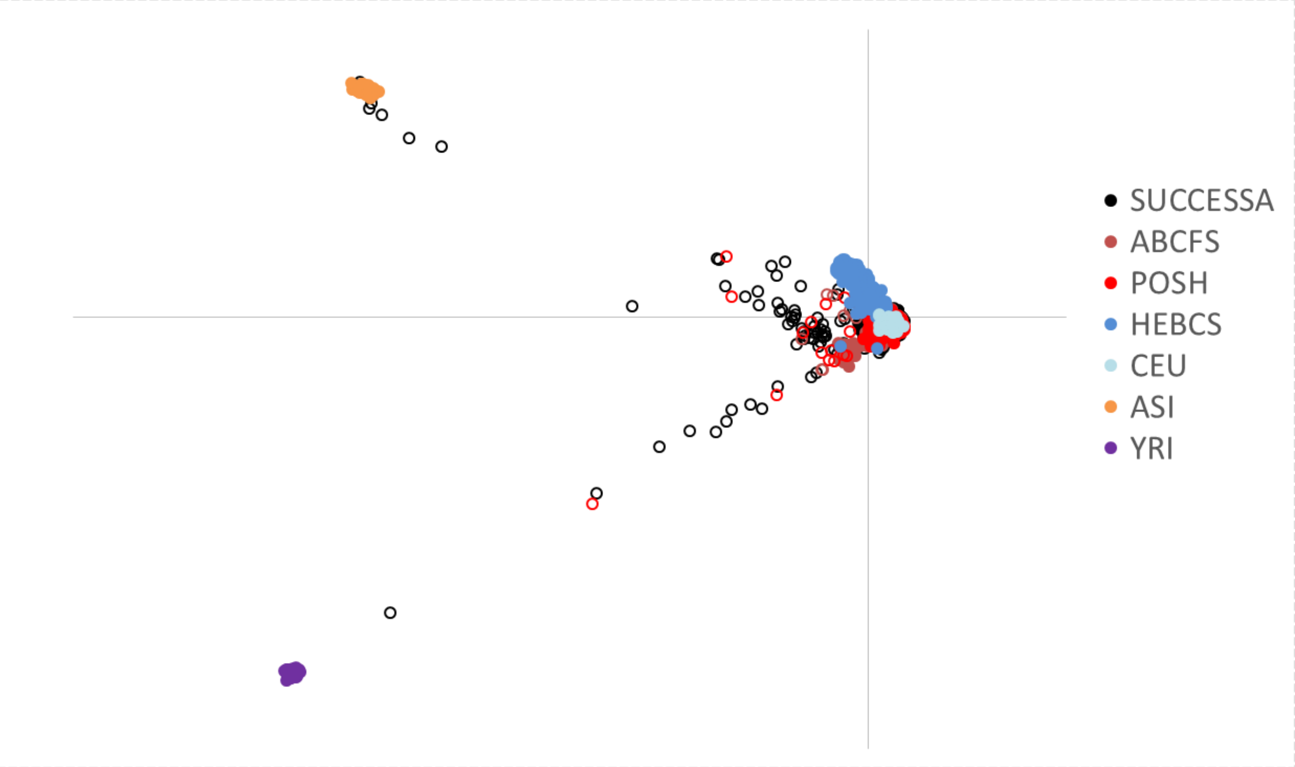

Cases from ABCFS, HEBCS, POSH and SUCCESS-A are plotted alongside HapMap reference populations from Utah with northern and western European ancestry (CEU), Japan and China (ASI) and Nigeria (YRI). Filled circles for ABCFS (●), HEBCS (●), POSH (●) and SUCCESS-A (●) indicate patients used for analysis which form a single tight cluster that overlaps with the CEU reference. Empty circles indicate samples that were excluded from analysis (n=123) because their genotypes did not concur with a European ancestry.

Supplementary Figure 2. Estimated power to detect SNPs influencing survival

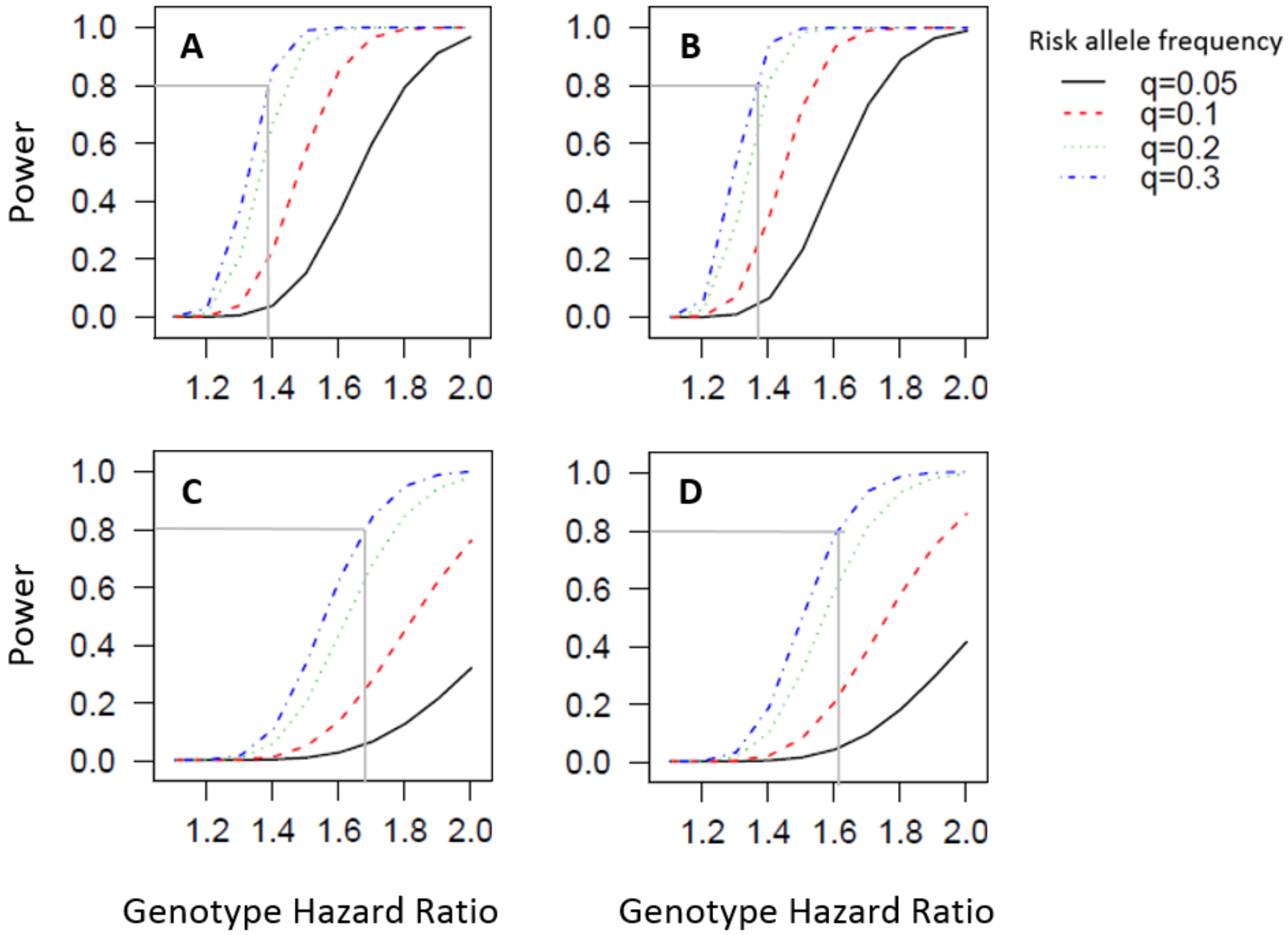

Power calculations for overall survival and disease-free survival for all patients at stage-1, plots A and B respectively, and a subset of stage-1 patients with early onset, plots C and D respectively.

Supplementary Figure 3: QQ plots for overall survival

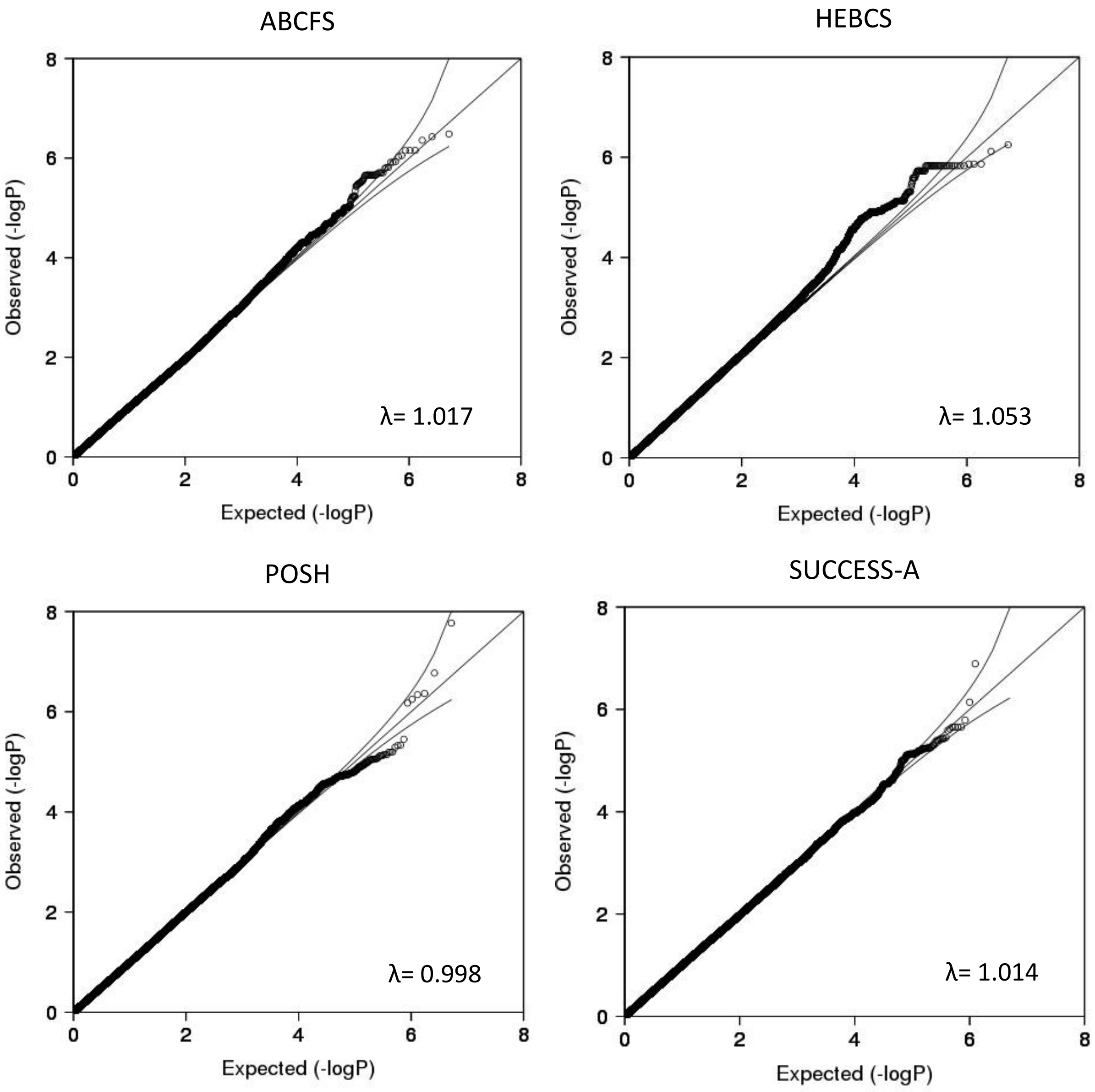

QQ plots showing the relationship between observed (Y-axis) and expected (X-axis) *P*-values (both expressed as – $\log_{10}$P). The *P*-values are for overall survival adjusted for ER status (COX regression, GenABEL) of the imputed data. The number of SNPs and cases in each cohort were; ABCFS 5,150,529 SNPs and 202 cases, HEBCS 5,395,529 SNPs and 798 cases, POSH 5,196,034 SNPs and 556 cases and SUCCESS-A 5,006,474 SNPs and 3183 cases. The region between the curved lines indicates bootstrapped 95% confidence intervals. The diagonal black line indicates expected results under null hypothesis. Genomic inflation factor (λ).

Supplementary Figure 4: QQ plots for disease-free survival

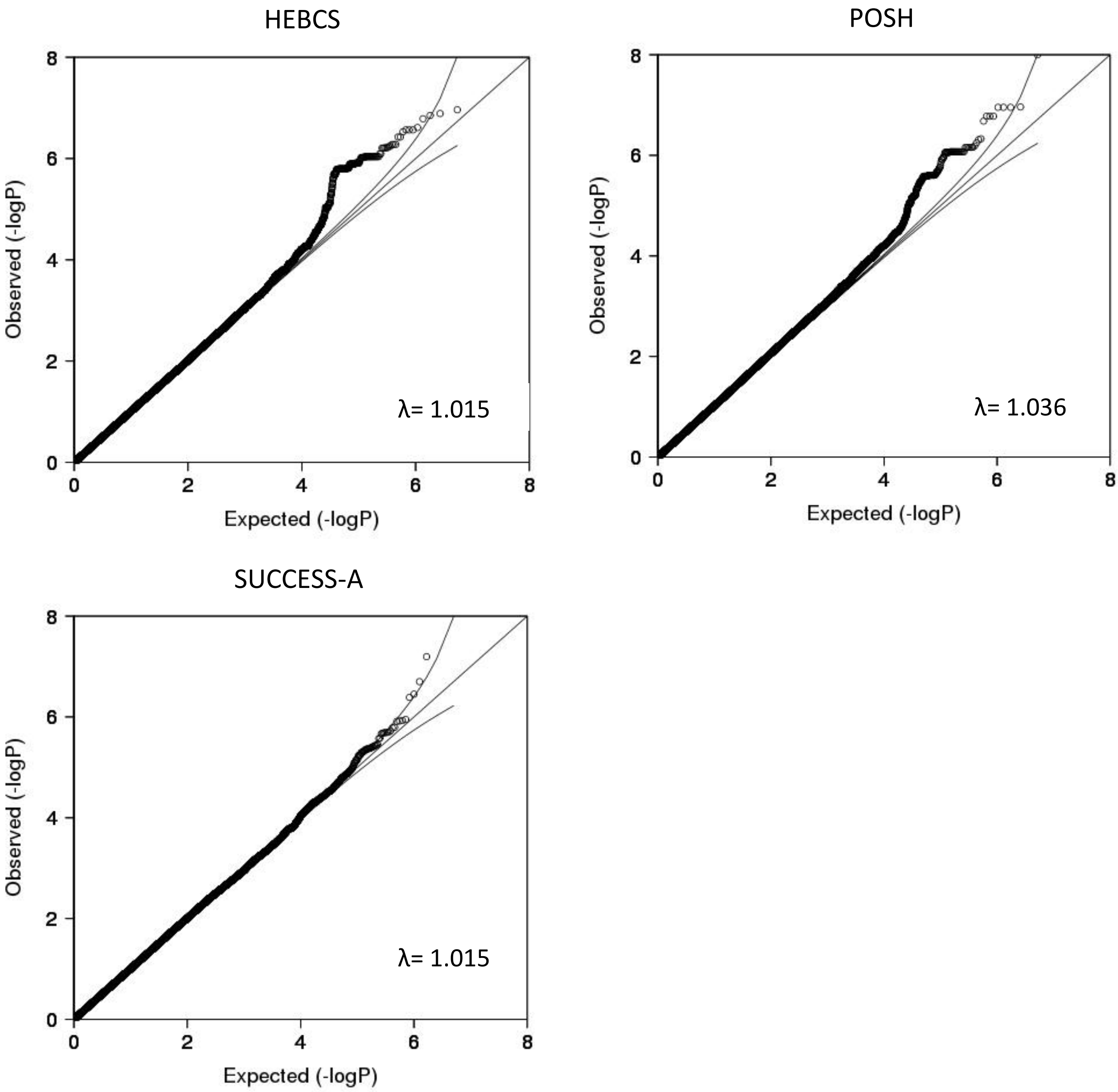

QQ plots for disease-free survival analysis adjusted for ER status (COX regression, GenABEL) of the imputed data. The number of SNPs and cases in each cohort were; HEBCS 5,395,529 SNPs and 798 cases, POSH 5,196,034 SNPs and 556 cases and SUCCESS-A 5,006,474 SNPs and 3183 cases. Genomic inflation factor (λ).

Supplementary Figure 5: QQ plots for overall survival and disease-free survival in patients with early onset

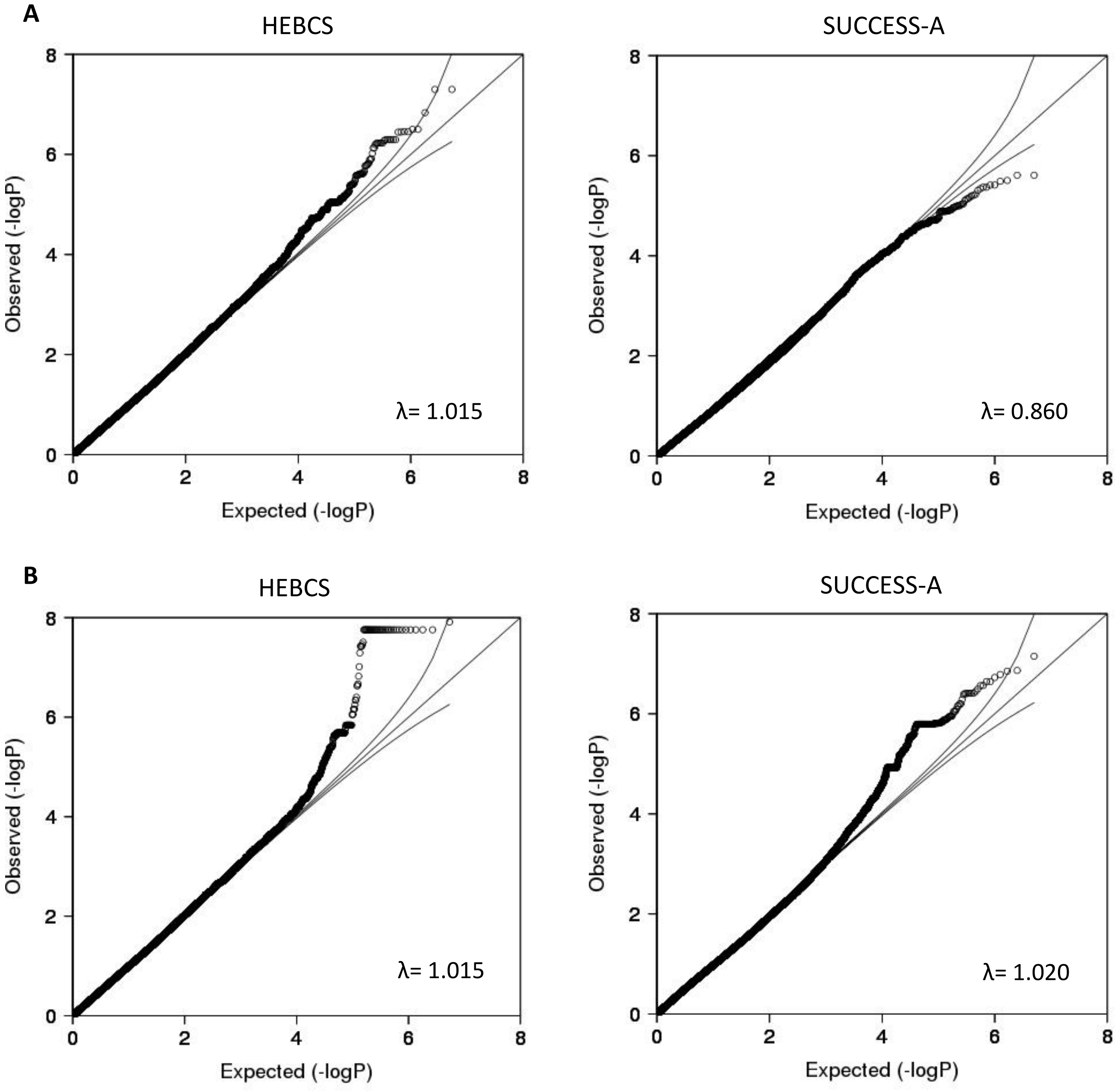

QQ plots for overall survival (A) and disease-free survival (B) with adjustment for ER status (COX regression, GenABEL). QQ plots for the POSH cohort are shown in Supplementary Figures 3 (OS) and 4 (DFS). The number of SNPs and cases in each cohort were; HEBCS 5,395,529 SNPs and 119 cases, POSH 5,196,034 SNPs and 556 cases and SUCCESS-A 5,006,474 SNPs and 337 cases. Genomic inflation factor (λ).

Supplementary Figure 6. Relationship between rs715212 and expression of AREG

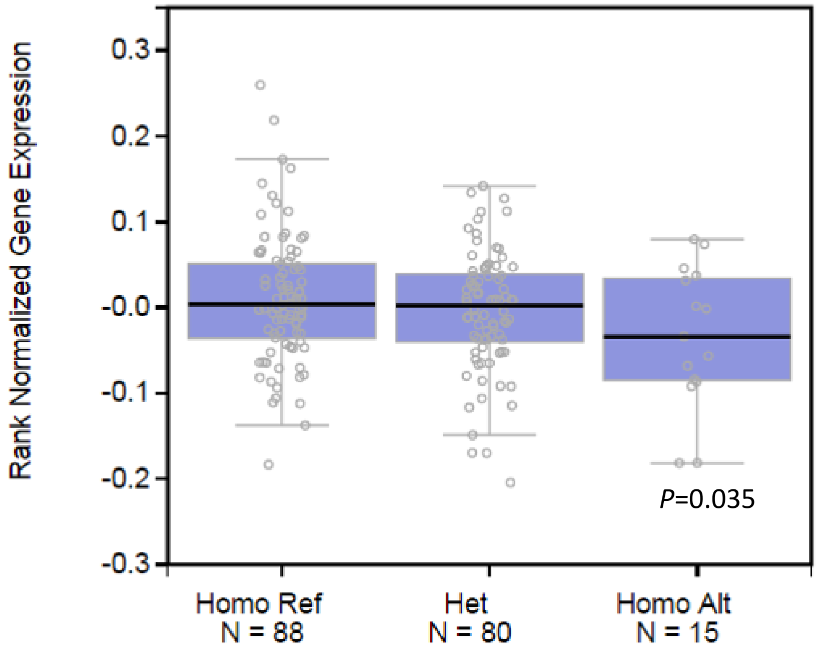

Expression quantitative trait locus box plot for the relationship between rs715212 and AREG expression in breast tissue.

Supplementary Figure 7. Kaplan-Meir survival plots for rs410155 in cases with triple negative breast cancer

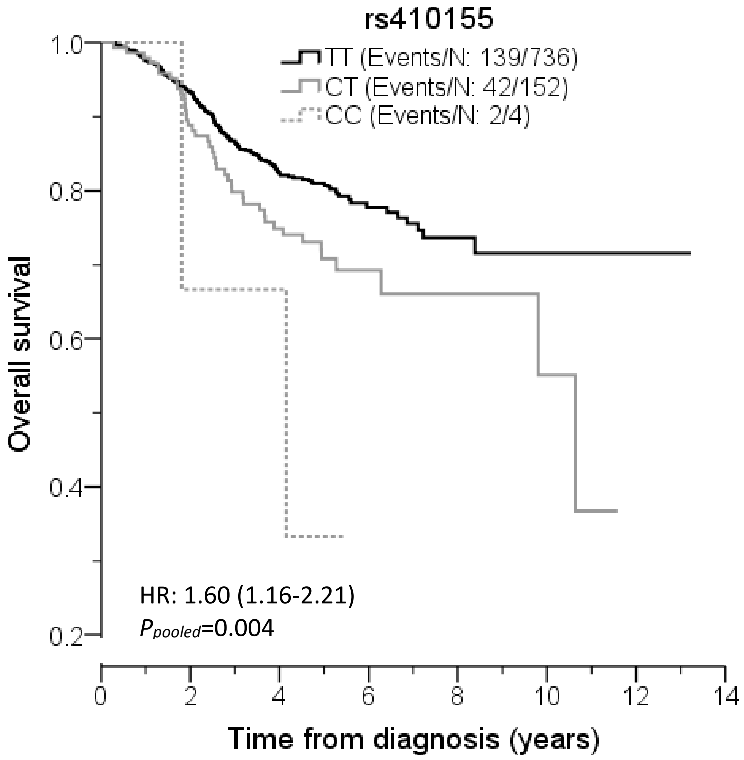

Kaplan-Meier plots from univariate analysis of rs410155 associated with overall survival (OS) in cases with triple negative breast cancer (TNBC). Data from four cohorts (HEBCS, POSH stages 1 and 2 and SUCCESS-A) were pooled. HR: hazard ratio with 95% confidence interval.
