# Supplementary material for: Germline variation in ADAMTSL1 is associated with prognosis following breast cancer treatment in young women
Source: Nat Commun. 2017 Nov 21;8:1632. doi: 10.1038/s41467-017-01775-y (PMC5696339; doi:10.1038/s41467-017-01775-y)
Supplement: Supplementary file 3 — Description of Additional Supplementary Files [file 41467_2017_1775_MOESM3_ESM.pdf]

Description of Additional Supplementary Files

File Name: Supplementary Data 1
Description: Selection criteria and results from all survival and meta-analyses for the 95 SNPs that were selected for genotyping at stage-2. Cells highlighted in dark green have P

File Name: Supplementary Data 2
Description: Results from stage 1 meta-analysis for previously published associations with breast cancer survival. Published associations with $p \leq 0.05$. OS: Overall survival, DFS: Disease-free survival, DDFS: Distant disease-free survival, EFS: Event-free survival, BCS: Breast cancer-specific survival, ER: Oestrogen receptor status. NA: Not available in the current study. *These estimates are taken from Additional File 3 of Pirie et al (2015)

File Name: Supplementary Data 3
Description: Functional inference of SNPs with significant replication. The index SNP and SNPs in LD ($r2 > 0.2$ in 1000 Genomes EUR Phase 1 data) are annotated with respect to histone marks, DNAse hypersensitivity, proteins bound and motifs changed. In addition, SNPs are annotated according to conservation metrics from combined annotation dependent depletion (CADD) scores and genomic evolutionary rate profiling (GERP) and transcription factor occupancy from RegulomeDB. RegulomeDB scores: 3a TF binding + any motif + DNase peak; 4, TF binding + DNase peak; 5, TF binding or Dnase peak; 6, other binding or Dnase peak. P-values for expression quantitative trait locus (eQTL) analysis are from the Genotype-Tissue Expression (GTEx) portal (V6, dbGaP Accession phs000424.v6.p1) for breast mammary tissue in 183 samples with genotype data.
